# Retraction: Emergence of Asian endemic begomoviruses as a pandemic threat

**DOI:** 10.3389/fpls.2023.1237555

**Published:** 2023-06-19

**Authors:** Frontiers Editorial Office

Following publication, concerns were raised to the journal regarding factual inaccuracies throughout the Review article text and errors in the data presented in **Figure 3**. Many of the references cited are also considered to be inappropriate or outdated. An investigation was conducted in accordance with Frontiers’ and the Committee on Publication Ethics (COPE) guidelines, whereby it was confirmed that the complaints were valid.

This retraction was approved by the Chief Editors of Frontiers in Plant Science and the Chief Executive Editor of Frontiers. The authors agree to the retraction.

